# Feasibility and acceptability of SEPA+PrEP: An HIV prevention intervention to increase PrEP knowledge, initiation, and persistence among cisgender heterosexual Hispanic women

**DOI:** 10.1371/journal.pone.0296080

**Published:** 2024-01-02

**Authors:** Rosina Cianelli, Joseph P. De Santis, Giovanna C. De Oliveira, Jose G. Castro, Evelyn Iriarte, María José Baeza, Sophia O. Thomas, Natalia Villegas, Nilda Peragallo-Montano

**Affiliations:** 1 School of Nursing and Health Studies, University of Miami, Coral Gables, Florida, United States of America; 2 Escuela de Enfermería, Pontificia Universidad Católica de Chile, Santiago, Chile; 3 Miller School of Medicine, University of Miami, Miami, Florida, United States of America; 4 College of Nursing, University of Colorado, Aurora, Colorado, United States of America; 5 School of Nursing, The University of North Carolina at Chapel Hill, Chapel Hill, North Carolina, United States of America; ☯ These authors contributed equally to this work; Nova Southeastern University / Yarmouk University, UNITED STATES

## Abstract

The HIV epidemic disproportionately affects Hispanics in the U.S., with Hispanic women (HW) accounting for 18% of new HIV diagnoses in 2019 despite comprising only 16% of the female population. The imbalance of power related to cultural values and HW’s lack of knowledge and low perception of risk for HIV interferes with prevention efforts (e.g., condom use, HIV testing, and Pre-Exposure Prophylaxis [PrEP]). It is estimated that in 2019, only 10% of women in the U.S. who could benefit from PrEP were given prescriptions for it. This number is estimated to be significantly lower among HW. PrEP is highly effective for preventing HIV, reducing the risk of acquiring HIV from sexual activity by about 99%. To respond to this need, we developed SEPA+PrEP, a biobehavioral HIV prevention intervention that adapted and integrated SEPA (Salud/Health, Educación/Education, Prevención/Prevention, Autocuidado/Self-Care), an empirically validated behavioral HIV prevention intervention, with the evidence-based biomedical strategy of PrEP. This study aimed to investigate the feasibility and acceptability of SEPA+PrEP among cisgender heterosexual Hispanic women (HW). We used a mixed methods approach to gather data from 44 HW living in the City of Homestead and its surrounding communities in Miami-Dade County, Florida. None of the participants knew about PrEP prior to participating in the study, and the majority (70.5%, *n* = 23) had not used condoms when engaging in vaginal sex during the previous three months. Overall, study results suggest that SEPA+PrEP is an acceptable and feasible intervention to prevent HIV among HW, with a focus on PrEP knowledge, initiation, and maintenance.

## Introduction

Despite recent progress, HIV continues to affect Hispanics in the U.S. disproportionately [1, 2]. In 2019, Hispanic women (HW) made up approximately 16% of the female population in the U.S. but accounted for 18% of new diagnoses of HIV infection, a rate that is four times higher than the rate for non-Hispanic White women (5.3 vs. 1.7 per 100,000) [2, 3]. Further, HW are three times more likely to die from HIV infection than are non-Hispanic White women (2.4 vs. 0.8 per 100,000) [4, 5]. Notably, 89% of HW acquired HIV through heterosexual sexual activity [4, 6, 7]. Sexual HIV risk behaviors, social determinants of health (SDoH), and cultural norms impact HIV prevention efforts among HW, increasing the risk for infection [8, 9].

Despite significant progress in HIV prevention, HW continue to face multiple SDoH that increase their risk for HIV, such as low income, lack of health insurance, low educational attainment, language barriers, and cultural norms and socio-environmental factors that limit access to HIV education and prevention services. These and other SDoH characterize the cisgender (i.e., a person whose gender identity aligns with the sex they were assigned at birth) heterosexual HW residing in the Miami-Dade County target communities where this study was conducted (i.e., the City of Homestead and its surrounding communities) [7, 9–13]. These factors contribute to persistent disparities in HIV incidence and prevalence among HW and highlight the need for innovative and comprehensive approaches to HIV prevention that consider the unique needs and circumstances of this population.

Among HW, HIV risk factors such as their partner’s risky sexual behaviors (e.g., sexual activity under the influence of alcohol, multiple partners, unprotected sex/no or lower condom use) and traditional gender roles negatively impact HIV prevention efforts [14, 15]. Heterosexual relationships may exhibit an imbalance of power linked to two Hispanic cultural values: machismo and marianismo. Machismo encourages Hispanic men to have multiple sexual partners and to engage in risky sexual behaviors, which increases their likelihood of contracting HIV. Marianismo promotes sexual passivity and encourages HW to accept men’s decisions on sexual matters (e.g., infidelity, condom use) [14, 16–18]. HW are often unaware of their HIV status [4, 5]. Further, HW’s lack of knowledge about and low perception of risk for HIV interferes with prevention efforts (e.g., condom use, HIV testing, Pre-Exposure Prophylaxis [PrEP] knowledge, initiation, and persistence) [2, 9, 19, 20].

A recent report, “Ending the HIV Epidemic (EHE) in the United States: A Plan for America” describes a federal effort to reduce new HIV infections by 90% by 2030; one EHE initiative aims to increase prevention efforts such as PrEP use and HIV testing [7]. The CDC estimates that in 2019, only 10% of women in the U.S. who could benefit from PrEP were given prescriptions for it [7]. This number is estimated to be significantly lower among HW [21, 22]. PrEP is highly effective for preventing HIV, reducing the risk of acquiring HIV from sexual activity by about 99% when taken as prescribed [23]. However, despite PrEP’s established efficacy and availability for over 10 years in the U.S., it is clear that PrEP is not being used by HW who might benefit from it as part of an HIV prevention plan [2, 22].

These disparities highlight the urgency for HIV prevention interventions for HW that incorporate PrEP. To respond to this need, we developed SEPA+PrEP, a biobehavioral HIV prevention intervention that adapted and integrated SEPA (Salud/Health, Educación/Education, Prevención/Prevention, Autocuidado/Self-Care), an empirically validated behavioral HIV prevention intervention, with the evidence-based biomedical strategy of PrEP. This study responds to and addresses the need to increase PrEP uptake among HW by investigating the feasibility and acceptability of SEPA+PrEP.

## Methods

### Research design

We used a mixed methods approach to explore the feasibility and acceptability of SEPA+PrEP among HW. More specifically, a triangulation mixed method design was used. This design collects both quantitative and qualitative data and uses both types of data for the interpretation of results [24].

### Study setting

We conducted the study in the City of Homestead and its surrounding communities in Miami-Dade County, Florida. Hispanics predominate (86%) in this geographic area; more than a quarter of the population (26.8%) is foreign-born and more than half of residents (50.9%) speak a language other than English at home [25]. We recruited participants through three community organizations, from public places, and online. The research team and members of these organizations conducted community outreach to identify potential participants at public sites where HW often gather and/or work (e.g., grocery stores, churches, restaurants). We distributed flyers in Hispanic neighborhoods, asked community members to spread the word about the study, and advertised the study through Facebook ads.

### Participants

A convenience sample of 44 HW participated in this study. Eligibility criteria included a) self-identification as a cisgender heterosexual Hispanic woman (HW); b) residence in the City of Homestead and its surrounding communities in Miami-Dade County, Florida; c) ages 18–49; d) speak and read Spanish; e) HIV antibody negative or unknown HIV serostatus; f) have engaged in sexual activity with a man in the last 6 months; and g) have at least one sexual risk factor for HIV (e.g., more than one sexual partner in the last 6 months; unprotected sex; substance use before or during condomless sex with sexual partner[s]). The University of Miami Institutional Review Board approved the study in August, 2020, and all participants provided verbal consent prior to completing the study assessments. Only the study PI has access to the linking list that allows identification of the participants. We recruited participants from February to September, 2021, and paid them $35 for their participation in each assessment, SEPA+PrEP or focus group session to compensate them for their time and transportation expenses.

### SEPA+PrEP intervention

SEPA+PrEP (Salud/Health, Educación/Education, Prevención/Prevention, Autocuidado/Self-Care + Pre-Exposure Prophylaxis) is an adapted, culturally appropriate, biobehavioral HIV prevention intervention for HW ages 18–49 to increase knowledge about, initiation, and persistence of PrEP, HIV testing, and condom use. SEPA+PrEP adapts SEPA, an empirically validated prevention intervention for HW ages 18–50 that is grounded in two theoretical models: Bandura’s Social Cognitive Model of Behavioral Change [26] and Freire’s Pedagogy [27].

Bandura’s Social Cognitive Model views the performance of a behavior as a function of outcome expectancies and self-efficacy (i.e., confidence in one’s ability to perform the behavior). Self-efficacy is built through rehearsal, role modeling, and support for the specific new behavior. Behavioral change (e.g., condom use, HIV testing, PrEP) requires interactions between the person, behavior, and others in the environment. Interventions to build self-efficacy and promote behavioral change must be closely tied to the target group’s specific cultural-social environment [26].

Freire’s pedagogy focuses on collaborative learning; it recognizes the impact and power of education and fosters greater learning through collaboration, communication, creativity, and critical thinking. Freire’s pedagogy drives the delivery of SEPA’s content and contextual learning by a) establishing the importance of every individual in the group as a contributor to the knowledge and skills that are generated during a session, and b) providing an atmosphere that fosters collaboration and encourages participants to engage in discussion and activities [27].

SEPA has been found to be effective in preventing or reducing sexual risk behaviors and depressive symptoms in randomized trials [9–11]. However, despite its efficacy in reducing HIV risk behaviors, SEPA does not include PrEP-related content and activities. Therefore, we adapted SEPA to create SEPA+PrEP as part of a Center for AIDS Research (CFAR) grant [28]. The adaptation process involved the participation of a Community Advisory Board (CAB), an expert panel, local healthcare providers, community agencies, researchers who developed SEPA, and HW that the intervention seeks to serve.

The ADAPT-ITT model guided our adaptation, ensuring this process was systematic and rigorous—an essential condition to maintain the original intervention’s fidelity [29]. ADAPT-ITT facilitates the adaptation of an existing evidence-based intervention (EBI) in a novel situation without changing, competing with or contradicting the EBI’s core elements or theoretical underpinnings [29]. Our adaptation of SEPA into SEPA+PrEP focused primarily on content, new activities, and delivery methods relevant to our HW target population (Table 1).

**Table 1:** Summary of SEPA+PrEP session topics and activities.

Session Number	Session Name	Content	SEPA+PrEP Activities
1.	HIV in the Hispanic Community	• Basic facts about HIV among Hispanic women • Soap opera: “Sin Verguenza/ Without Shame” • What are HIV and AIDS? • How is HIV transmitted? • Introduction to Hispanic cultural values, beliefs, and traditions • Introduction to HIV prevention with an emphasis on PrEP	a) Introduction and group commitments^a^ b) Soap opera discussion^a^ c) Facilitated small group discussions^a^^,^^b^^,^^c^ d) Interactive activities^a^^,^^b^^,^^c^ e) Home-based activities^a^^,^^b^^,^^c^ f) Session evaluation^a^^,^^b^^,^^c^ g) Skill-building exercises to promote condom use^b^ h) Skill-building activities to communicate more effectively with partners and family^c^ i) Role-playing^c^ j) Intervention evaluation^c^ k) Reflection and commitments^c^ l) Certificate in recognition of SEPA+PrEP completion^c^
2.	HIV Prevention	• Rumors, myths, and beliefs surrounding HIV/AIDS• STIs and their role in HIV• HIV prevention • PrEP • HIV testing • Condom use • How Hispanic cultural values and traditions are related to HIV prevention	
3.	Partner Communication & Negotiation/ Intimate Partner Violence Prevention in the Context of HIV	• Partner communication and negotiation strategies to prevent HIV • Self-esteem in the context of partner communication and negotiation • Intimate partner violence prevention in the context of HIV • HIV prevention discussion with an emphasis on review of PrEP content	

*Note*. AIDS = acquired immunodeficiency syndrome; HIV = human immunodeficiency virus

STI = sexually transmitted infections; PrEP = Pre-Exposure Prophylaxis

^a^ Activity conducted in Session 1

^b^ Activity conducted in Session 2

^c^ Activity conducted in Session 3

We used a Community-Based Participatory Research (CBPR) approach [30] to collect information to conduct the adaptation. This adaptation process is aligned with literature indicating that the adaptation of effective interventions is efficient and relevant to address the needs of specific populations such as HW [30].

#### Community Advisory Board (CAB)

A 15-member Community Advisory Board (CAB) met via Zoom® once per month for 10 months to inform and guide the project about HW’s HIV prevention needs; discussion focused on PrEP and on potential adaptations of SEPA into SEPA+PrEP. The CAB was comprised of community members, including representatives of community organizations, healthcare providers, community leaders, and HW from the community. We identified CAB members in collaboration with community organizations. A research team member provided all CAB members with 4 hours of training and discussion on HIV prevention and PrEP.

#### Expert panel

A panel of nine content experts provided recommendations to help guide the adaptation of SEPA into SEPA+PrEP. The panel was comprised of healthcare providers, researchers, and faculty with expertise in HW, HIV prevention, and intervention development.

#### Healthcare providers

We interviewed seven healthcare providers who work with HW. The healthcare providers offered recommendations for HIV prevention, PrEP, and prevention intervention elements that should be considered when adapting SEPA into SEPA+PrEP.

#### Cisgender heterosexual Hispanic women (HW)

We conducted a focus group with ten cisgender heterosexual HW from the target community. We recruited the focus group members using the same methods that we used to recruit the HW who participated in the SEPA+PrEP groups; both groups met the same inclusion criteria. The focus group met for one hour via Zoom®. The women talked about HIV awareness in the community. They noted that HW overall tend to have limited knowledge about HIV and affirmed that it was important to include the content on Hispanic cultural norms, HIV, inclusion of PrEP, and use of female condoms in SEPA+PrEP. The women also reported that because Hispanic men usually do not want to use condoms, PrEP could provide HW with more control over their sexual health.

#### SEPA+PrEP facilitators

Working closely with our community organization partners, we selected three bilingual Hispanic women from the community to facilitate the SEPA+PrEP intervention. Prior to initiating the SEPA+PrEP intervention, the facilitators received 12 hours of training conducted by the study PI and Co-I: the same 4 hours of training on HIV and PrEP that was offered to the CAB, and 8 hours of training on how to implement the 3-session SEPA+PrEP protocol.

#### SEPA+PrEP sessions

Facilitators delivered the SEPA+PrEP intervention in three weekly 2-hour sessions with small groups of 6–10 participants. Sessions were held in a private space at a community organization and included facilitated discussions, interactive feedback, role-playing, role modeling, rehearsal of communication skills, skill-building activities, and social interaction. The content and activities were organized around three sessions: Session 1. HIV in the community; Session 2. HIV prevention strategies and PrEP; and Session 3. Communication and negotiation with the partner and intimate partner violence.

### Data collection

We conducted all study activities in Spanish, the participants’ preferred language. We interviewed participants via phone call or Zoom® prior to their participation in the SEPA+PrEP intervention using a secure web-based software (Qualtrics) to document responses electronically. Following each of the three SEPA+PrEP sessions, participants responded to a paper-pencil questionnaire consisting of 4 open-ended questions about the session. At the end of the third session, the HW also responded to a feasibility and acceptability paper-pencil questionnaire. We also invited 25 of the 44 women who completed the intervention to participate in one of four post-intervention focus groups.

#### Study measures

*Before participating in the SEPA+ PrEP intervention*. We assessed socio-demographic variables using a 17-item questionnaire to collect descriptive information about age, current relationship status, housing situation (with whom they lived), length of time the participant had been living with her partner (if she was living with a partner), number of children, religion, educational level, main occupation and type of employment, income in U.S. dollars, and health insurance status. Participants also responded to five questions about HIV prevention: (1) *Have you been tested for HIV*? (yes, no); (2) *Why did you get the HIV test*? (tested during prenatal care; recommended by health care provider; ability to get results in same day; ability to maintain confidentiality of results); (3) *How concerned are you about getting HIV/AIDS*? (not at all, somewhat, extremely); (4) *How often did you use condoms during vaginal sex with your main partner in the last 3 months*? (never, less than half of the time, more than half of the time, always, not applicable); and (5) *Have you heard about HIV Pre-Exposure Prophylaxis (PrEP*?*)* (yes, no).

*During participation in the SEPA+ PrEP intervention*. We assessed feasibility—defined as the capacity of the intervention to be successfully implemented—through session attendance, retention rates, and completion of session feedback [31]. Our goal was to meet a threshold of at least 80% attendance at each of the three sessions. We assessed acceptability—defined as satisfaction with the intervention and the degree to which it met expectations—in two ways. At the end of each SEPA+PrEP session, participants responded to four open-ended questions: *What did you most like about this session*?; *What did you least like about this session*?*; Is there a topic you would like to repeat or clarify*?*; Do you have other comments about the session*? At the end of session three, participants completed a 14-item acceptability questionnaire that was previously used with Hispanic communities [31]. The 11 multiple-selection and three open-ended questions assessed satisfaction with SEPA+PrEP’s content and activities, clarity and comfort, the relationship between facilitators and participants, and expectations about the intervention. The questionnaire also elicited suggestions about how to improve the intervention [31].

*After participating in the SEPA+ PrEP intervention*. We invited 25 of the 44 participants who completed all three SEPA+PrEP sessions to participate in one of four 60-minutes focus groups. Collecting qualitative data through the focus groups gave us a deeper understanding of the HW’s intervention experience and allowed us to assign meaning to the quantitative data. Focus groups were conducted via Zoom® and audio-recorded. We developed a semi-structured interview consisting of open-ended questions based on the literature and on our experience working with HW (e.g., *What do you think about SEPA+PrEP*?, *Do you think SEPA+PrEP responds to community needs*?, *Do you think SEPA+PrEP should be implemented with other groups of people*?). We ensured consistency by using the same interview guide for the four focus groups. Audio recordings were transcribed verbatim and stored in a locked office, with digital files saved on password-protected computers.

### Data analysis

We conducted quantitative analyses using the Statistical Package for Social Sciences (SPSS) version 28.0 and used descriptive statistics to analyze the quantitative data. To better understand the participants’ experience, we used conventional content analysis to identify qualitative content in the focus group transcripts that reflected the participants’ quantitative responses [32]. Three research team members used a parallel analysis approach to identify the content; this approach is used when both quantitative and qualitative data are collected in the same study. In parallel analysis, both types of data are viewed as contributing equally to the study’s results. Although the quantitative and qualitative data are analyzed separately or independently, results are interpreted in an integrated manner [24] that identifies whether and how the two types of data inform and reinforce each other.

## Results

### Socio-demographic characteristics

A total of 44 HW with a mean age of 37 years (*SD* = 8.5; range 18–49) completed the baseline questionnaire (Table 2). Most participants were born outside of the U.S. (90.9%, *n* = 40), with the majority coming from Mexico (52.3%, *n* = 23) and Guatemala (18.2%, *n* = 8); most 93.2% (*n* = 41) preferred to speak Spanish in their daily life activities. A majority of the participants were either married (43.2%, *n* = 19) or living with a male partner (not married) (36.4%, *n* = 16). Participants had completed a mean of 9.3 years of education (*SD* = 4.0, range 0–16) and had an average monthly family income of $1,989.3 (*SD* = $934.1; range $400-$4,000); 65.9% (*n* = 29) were employed. Almost two-thirds (63.6%, *n* = 28) reported not having health insurance.

**Table 2:** SEPA+PrEP participants’ socio-demographic characteristics (N = 44).

Variables	*n* (%) or *M* (*SD*; range)
**Age (in years)**	37 (8.5; 18–49)
**Relationship status**	
Married	19 (43.2)
Living with a male partner (not married)	16 (36.4)
Single	7 (15.9)
Divorced	2 (4.5)
**Years of education**	9.3 (4.0; 0–16)
**Employment status**	
Employed	29 (65.9)
Not employed	18 (38.3)
**Main occupation**	
Nurseries (plant care)	9 (31.0)
Housekeeping	7 (9.1)
Homemaker	5 (11.4)
Other (e.g., student, teacher, social worker)	23 (52.3)
**Per capita income**	$1,989.3 ($934.1; $400-$4,000)
**Preferred language**	
Spanish	41 (93.2)
English	3 (6.8)
**Place of birth**	
Mexico	23 (52.3)
Guatemala	8 (18.2)
El Salvador	5 (11.4)
United States	4 (9.1)
Dominican Republic	2 (4.5)
Colombia	1 (2.3)
Honduras	1 (2.3)
**Years living in the U.S.**	16.1 (8.4; 0–31)
**Has health insurance (no)**	28 (63.6)

*Note*. *M* = mean; *n* = number; *SD* = standard deviation.

### HIV prevention behaviors prior to participation

Almost all of the participants (88.6%; *n* = 39), reported having been tested for HIV at least once; more than half (52.3%, *n* = 23) had been tested during prenatal care (Table 3). When asked whether they had used a condom during vaginal sex during the previous three months, a majority (70.5%, *n* = 31) reported never having used a condom, while only five (11.4%) reported always having used a condom. In addition, 56.8% (*n* = 25) of participants reported that they were not concerned about acquiring HIV. None of the participants were aware of PrEP before the intervention and all of them described the intervention as an excellent resource that had helped them learn about PrEP.

*In my case*, *it was very good*. *It was new information because I didn’t know*, *for example*, *about the PrEP pill*. *I didn’t have any information*, *I didn’t even know it existed*. *And through the sessions I learned what it is for*, *and I gained more information about all of that*.[All participant quotes were translated from Spanish by the authors.]

**Table 3:** SEPA+PrEP HIV prevention behaviors prior to participation.

Questions	*n* (%) or *M* (*SD*; range)
Number of times tested for HIV	4.2 (5.4; 0–30)
Ever tested for HIV (yes)	39 (88.6)
Reasons for testing	
Tested during prenatal care	23 (52.3)
Recommended by health care provider	17 (38.6)
Ability to get results in same day	7 (15.9)
Ability to maintain confidentiality of results	6 (13.6)
Condom use with vaginal sex in previous 3 months	
Never	31 (70.5)
Less than half of the time	3 (6.8)
Half of the time	3 (6.8)
More than half of the time	2 (4.5)
Always	5 (11.4)
Concerned about becoming infected with HIV (no)	25 (56.8)

*Note*. *M* = mean; *n* = number; *SD* = standard deviation.

### Intervention feasibility and acceptability

All participants (*n* = 44; 100%) attended all three sessions, successfully completed the SEPA+PrEP intervention, and provided feedback at the end of each session.

We assessed acceptability by asking participants to respond to four open-ended questions at the end of each session and to complete the 14-item acceptability questionnaire at the end of session three [31]. Results integrated participants’ responses to the four questions, the acceptability questionnaire, and the focus group qualitative data. Quantitative results supporting SEPA+PrEP’s acceptability can be found in Table 4.

**Table 4:** Acceptability of the SEPA+PrEP intervention.

Questions	*n* (%) or *M* (*SD*; range)
Understanding of the information deliveredª	4.3 (1.0; 1–5)
High/Very high	39 (88.6)
Neutral	3 (6.8)
Low/Very low	2 (2.5)
Satisfaction with the interventionª	4.9 (0.3; 4–5)
Satisfied/Very satisfied	44 (100.0)
Neutral	0 (0)
Dissatisfied/Very dissatisfied	0 (0)
Degree of comfort with the methodologies usedª	4.4 (1.0; 1–5)
High/Very high	39 (88.6)
Neutral	2 (4.5)
Low/Very low	3 (6.8)
Satisfaction with the facilitatorª*	4.9 (0.4; 3–5)
High/Very high	42 (97.7)
Neutral	1 (2.3)
Low/Very low	0 (0.0)
Satisfaction with the information providedª	4.9 (0.3; 4–5)
Satisfied/Very satisfied	44 (100.0)
Neutral	0 (0)
Dissatisfied/Very dissatisfied	0 (0)
Satisfaction with the intervention activitiesª	4.8 (0.4; 4–5)
Satisfied/Very satisfied	44 (100.0)
Neutral	0 (0)
Dissatisfied/Very dissatisfied	0 (0)
Inclusion of topics that are relevant for women like them^b^**	2.8 (0.5;1–3)
Yes	33 (78.6)
Neutral	8 (19.0)
No	1 (2.4)
Fulfillment of initial expectations^b^	2.9 (0.4; 1–3)
Yes	40 (90.9)
Partially	3 (6.8)
No	1 (2.3)
Likely to participate in similar programs^b^	3.0 (0.0; 3)
Yes	44 (100)
Neutral	0 (0)
No	0 (0)
Likely to recommend the intervention to friends^b^	3.0 (0.0; 3)
Yes	44 (100)
Neutral	0 (0)
No	0 (0)
Acceptability of an online version of the intervention^b^	1.8 (0.9; 1–3)
Yes	22 (50.0)
Neutral	9 (20.5)
No	13 (29.5)

*Note*.

*One participant did not answer this question.

** Two participants did not answer this question. *M* = mean; *n* = number; *SD* = standard deviation.

^a^ Range of possible scores = 1–5.

^b^ Range of possible scores = 1–3.

#### Satisfaction with the intervention

All participants (*n* = 44, 100%) reported a high level of satisfaction with the intervention content (*M* = 4.9, *SD* = 0.3, range = 4–5). In the focus groups, participants expressed satisfaction with the SEPA+PrEP intervention, noting that prior to the intervention they had been unfamiliar with most of the health content discussed and that the intervention increased awareness of personal risk perception.

*The days I was there with you* [during the SEPA+PrEP intervention*]*, *I learned a lot*. *And I also educated myself a lot because*, *in reality*, *I didn’t know anything about this*. *The truth is that it is very valuable information for me*. *Because I think that PrEP is very important*, *especially for married couples*, *because there are many women who are exposed to [the risk of] contracting diseases*. [All participant quotes were translated from Spanish by the authors.]

#### Understanding the information delivered

We asked participants to rate their understanding of the information delivered during the SEPA+PrEP sessions and scored their responses on a 5-point scale (1 = *very low*; 5 = *very high*). Most participants (*n* = 39, 88.6%) found the content "very highly/highly understandable," while 6.8% (*n* = 3) found the information "neutrally understandable" and 2.5% (*n* = 2) reported "low/very low" understanding (mean score = 4.30, *SD* = 1.0, range = 1–5). Focus group participants reported that they understood the content and expressed a desire for more opportunities to continue discussing HIV prevention and transmission. As one participant remarked, *"continue talking about HIV*, *to continue learning*.*"* Another participant stated:

*For me also, it was a learning experience. I didn’t have any information. How to check a condom. How to put it on correctly. How is AIDS transmitted? What is AIDS and what is HIV? All this information has helped me a lot because there are many things that I did not know*.

#### Methodologies used to enhance learning

We asked participants to rate their comfort level with the images and examples used during the SEPA+PrEP sessions *("How comfortable were you with the examples*, *images/pictures*, *and movies used during the SEPA+PrEP sessions*?*"*) and scored their responses on a 5-point scale (1 = *very low comfort*; 5 = *very high comfort*). Most participants (*n* = 39, 88.6%) reported a high or very high level of comfort with the content (*M* = 4.4., *SD* = 1.0, range = 1–5). Qualitative data indicated that the use of a movie was effective in enhancing learning. One participant noted: *“The movie we saw was super important to me*. *It caught my attention because you learn more through movies than by reading…with the film*, *you don’t forget the information*.*”* Another participant emphasized the importance of using realistic images to demonstrate the signs and symptoms of sexually transmitted infections in both men and women: *“I had not seen such realistic photos*. *Because that makes us much more aware*. *Well*, *it helps us to take care of ourselves*.*”*

#### Satisfaction with the facilitators

Participants also expressed high levels of satisfaction with the intervention facilitators (*"Please rate your satisfaction with the facilitator of the SEPA+PrEP sessions"*); we scored their answers on a 5-point scale (1 = *very satisfied*; 5 = *very dissatisfied*). Most participants (*n* = 42, 97.7%) reported high or very high satisfaction with the facilitators (*M* = 4.9, *SD* = 0.4, range = 3–5). One participant noted:

*[The facilitator] is from our community*, *and she speaks very well… She talked about her personal life*, *so we also opened up a little more*. *I’ve been in other talks where it’s more about the PowerPoint and it’s over… But she takes her time…to speak to you*. *And that*, *you know*, *I loved about her*.

#### Acceptance of new content and activities related to PrEP and HIV prevention options

A majority of participants (*n* = 33; 78.6%) reported that the SEPA+PrEP sessions covered topics and issues women believed were important to them. The focus group discussion reinforced this finding, suggesting that the SEPA+PrEP content and activities were congruent with the participants’ needs and concerns. The HW also emphasized the need to provide more women from the community the opportunity to participate in the intervention: *"It would be very good to continue spreading the word to nieces*, *sisters-in-law*, *relatives*, *so it reaches the ears of other people*."

Participants were highly accepting of the intervention’s content and activities, particularly information specific to PrEP. Several HW noted that PrEP is suitable for women like them or that they were thinking of using PrEP:

*I think that PrEP is very important for married couples*. *There are many women who are at risk of contracting diseases*. *I would say almost all of us*. *And what you said*, *about the doctor*, *that we could go to the doctor and tell him that I am a person who can use PrEP*. *A candidate*. *That was great for me*.

*Yes*, *I would use it*. *When I have an appointment*, *I’ll talk to my doctor about PrEP*. *I understand that insurance covers it with a prescription*. *And I’ll tell him to test me*. *I can take the injection or the pill*, *whatever they prescribe*.

Others were intrigued by the female condom: *“Another thing that I loved*: *the female condom fascinated me*.*”*

Although the participants accepted PrEP, they believed that several barriers prevented them from accessing PrEP—most notably, the medical prescription required: *"And this thing about PrEP*, *well*, *this also seems to me to be a good thing*, *but…it has to be prescribed by a doctor and it’s the only drawback that I see*.*"*

One participant noted that the lack of health insurance among women in the community could hinder their access to and use of PrEP: *“There’s also a problem for many of us who don’t have insurance*. *They should make it easier to obtain*, *like in clinics*, *because many of us can’t obtain it through insurance*.”

#### Participant comments about other aspects of the SEPA+PrEP intervention

*Building community*. Participants often mentioned the group nature of the intervention. They reported having a positive experience spending time with other women and giving each other feedback. As one participant described: *“That first class*, *we felt a little embarrassed*, *but after that you felt free*. *Among women*, *among*, *let’s say*, *family*, *to talk and interact and give our point of view and ask our questions*.*”*

Another participant emphasized that interacting with other women allowed them to gain greater knowledge by exchanging personal histories and viewpoints: *“All this for me was very important*, *because we learn from each other*, *and some tell their stories*. *And that’s important because there are times when that’s what it’s about*: *learning from others*.*”*

*Inclusion of other community members to break down cultural and gender scripts that place HW at risk for HIV*. Participants believed involving more community members could help change social and cultural attitudes regarding sexuality, HIV prevention, and communication with partners. One participant emphasized the importance of targeting adolescents in school settings:

*Could you take it [SEPA+PrEP] to the schools*? *Yes*, *because not all mothers talk to their daughters*. *They aren’t very open due to the same patterns we bring from our countries*, *the culture we bring*. *They don’t talk about anything*, *they don’t talk about menstruation*, *they don’t talk about anything like that*, *so imagine*, *if it’s about sexuality*, *then even less*.

The focus groups also discussed including men as a strategy to decrease machismo:

*Not only the woman should be learning*, *but also the man*. *The man also has to go out and expel that machismo*, *as the other women here are saying*. *And that would be great for men*, *so they learn*, *and get rid of all those ideas they have about being machista*.

To involve more people, participants suggested outreach strategies to promote SEPA+PrEP in community locations such as schools, restaurants, and markets and to address SEPA+PrEP content in the community deliberately. One participant, for example, suggested going to work settings: “*I think*, *if you can find a place where people work*, *where you are allowed to go inside*.*”* Another recommended going to the local flea market: *“I think*, *at the flea market; there*, *you’ll have your table with the things you want to show and people will start coming*.*”*

## Discussion

Study results support the feasibility and acceptability of the SEPA+PrEP intervention among HW. Our findings underscore HW’s continued vulnerability to HIV and the need for effective and culturally-tailored prevention interventions that incorporate PrEP. Demographic data showed that overall, participants encountered many SDoH (e.g., limited formal education and household income, unemployment, lack of health insurance). The SEPA+PrEP intervention evaluated in this study represents an important step towards addressing these challenges.

### Community partnerships

The SEPA+PrEP intervention incorporated several features widely recognized as major advantages offered by Community-Based Participatory Research (CBPR), as reported by Rhodes et al. [29]. This study highlights the value of a long-standing CBPR partnership comprised of HW, community organization representatives, and academic researchers who worked together to adapt the SEPA HIV prevention intervention into SEPA+PrEP [29]. By adopting a community-driven approach, SEPA+PrEP was successful in addressing the needs of its vulnerable target population, which is often neglected in power-sharing approaches aimed at reducing HIV-related health disparities [30, 33].

This study also underscores the critical contribution of community facilitators in successfully delivering the intervention [33]. The HW expressed high levels of satisfaction with the intervention facilitators, underscoring the importance of community-driven approaches to HIV prevention among vulnerable populations. The peer facilitators—a culturally relevant form of leadership within Hispanic communities—were perceived positively by the HW and may have contributed to the acceptability and feasibility of the SEPA+PrEP intervention. These results support previous research emphasizing the value of involving community members in implementing health interventions, particularly among ethnic minorities who may experience unique challenges in accessing healthcare and HIV prevention services [34, 35].

### Participant engagement

Study findings also highlight the successful implementation of SEPA+PrEP as a multi-session intervention, with high participation, retention, and completion rates. The fact that all 44 participants attended every session shows a high level of engagement in and commitment to the intervention. Additionally, the fact that all participants completed the session feedback suggests that they were highly invested in helping to improve the intervention to benefit others in the community.

Participants reported that the SEPA+PrEP intervention met their initial expectations and provided appropriate content, activities, and features, demonstrating the feasibility of the intervention for HW. These results are particularly valuable, indicating that the HW felt comfortable with the intervention’s activities and group format. This positive outcome may have been due to the variety of features incorporated into the SEPA+PrEP sessions (e.g., movies, videos, images, examples, role-playing). Additionally, participant feedback was positive and will be instrumental in helping to improve SEPA+PrEP for future implementation.

### Increasing HIV-related knowledge

Study findings align with previous research that has reported low levels of awareness and knowledge about HIV prevention and transmission among minority women in the U.S. [36–39]. Most participants lacked the most basic information about these topics, and notably, no participants had any previous knowledge about PrEP. Without sufficient awareness and knowledge, HW may not be able to take proactive measures to prevent HIV (e.g., PrEP, HIV testing, female or male condom use). Therefore, this study highlights the critical need for targeted interventions such as SEPA+PrEP to improve HIV awareness and knowledge and to promote PrEP knowledge, initiation, and persistence among this population.

The SEPA+PrEP intervention used culturally-tailored approaches to address the lack of knowledge about HIV and PrEP among HW. The SEPA+PrEP sessions’ group-based approach created a safe and supportive environment for interpersonal learning, information sharing, and building cohesion among the participants. By sharing their experiences, participants realized that all other HW in the group also lacked knowledge about HIV infection. Attitudes towards HIV were discussed in non-judgmental and confidential small group settings. The SEPA+PrEP intervention was tailored to the Hispanic culture in the way that it addressed multiple areas of HIV prevention (e.g., PrEP, HIV testing, condom use), as well as communication and negotiation with partners, and intimate partner violence [9–11, 32, 38]. After learning about HIV and PrEP, most participants were open to discussing the potential use of PrEP.

### Cultural barriers to HIV prevention

Despite a high rate of previous HIV testing (88.6%), participants were unaware of the importance of regular testing and continued to engage in unprotected sex, revealing a low-risk perception of HIV infection. These behaviors are influenced by cultural factors such as machismo and marianismo, which discourage sexual communication with partners, normalize multiple sexual partners for men, and undervalue condom use in stable relationships. These findings align with previous research and suggest that interventions should tackle cultural factors to promote HIV prevention among HW effectively [21, 38, 39].

### Limitations

Study limitations include: 1) the self-report nature of data collected, which could influence outcomes; 2) the use of a convenience sample, which could have resulted in a sample not representative of all HW; and 3) conducting the study in the City of Homestead and surrounding communities in Miami-Dade County, Florida, The generalizability of our findings is limited by our unique study sample, which should not be viewed as representative of all Hispanic women in the U.S. We conducted the study in the City of Homestead and its surrounding communities in Miami-Dade County, an area with high numbers of foreign-born Hispanics whose first language is Spanish. Moreover, the cultural and social values of the HW in our study may be different from the HW born and raised in the U.S. Further, this is the first attempt to adapt and integrate SEPA with the evidence-based biomedical strategy of PrEP; additional research is needed to explore the potential impacts of this intervention.

## Conclusions

Overall, study results suggest that SEPA+PrEP, with a focus on PrEP knowledge, initiation and maintenance, is an acceptable and feasible intervention to prevent HIV among cisgender heterosexual HW whose demographic and social/cultural characteristics are similar to those of the HW who participated in our study. SEPA+PrEP demonstrates the importance of community engagement and culturally-tailored interventions in promoting health behaviors and reducing risk behaviors. The adaptation process followed rigorous guidelines, maintaining the original intervention’s core elements and theoretical framework while incorporating innovative PrEP-related content and activities. It is encouraging that many participants gained a greater understanding of HIV risk and HIV prevention strategies and were open to considering the use of PrEP after completing the intervention. Study findings also highlight the ongoing need for awareness of and education about HIV and PrEP among HW and the importance of addressing the SDoH that impact their access to HIV prevention services. Future research might explore the applicability of the SEPA+PrEP intervention in Hispanic communities with different demographic characteristics (e.g., greater numbers of HW born and raised in the U.S.). These studies can assess whether the intervention is similarly helpful, or whether adaptations are needed to meet the needs of HW and communities that may differ from our study sample and target community in significant ways.

## Supporting information

S1 Checklist (DOCX)

S1 Table Summary of SEPA+PrEP session topics and activities. (DOCX)

S2 Table SEPA+PrEP participants’ socio-demographic characteristics (N = 44). (DOCX)

S3 Table SEPA+PrEP HIV prevention behaviors prior to participation. (DOCX)

S4 Table Acceptability of the SEPA+PrEP intervention. (DOCX)

S1 File (PDF)

S1 Data (XLSX)
