# Supplementary material for: Feasibility and acceptability of SEPA+PrEP: An HIV prevention intervention to increase PrEP knowledge, initiation, and persistence among cisgender heterosexual Hispanic women
Source: PLoS One. 2024 Jan 2;19(1):e0296080. doi: 10.1371/journal.pone.0296080 (PMC10760780; doi:10.1371/journal.pone.0296080)
Supplement: S1 Table — (DOCX) [file pone.0296080.s002.docx]

**Table 1. Summary of SEPA+PrEP Session Topics and Activities.**

| **Session**  **Number** | **Session Name** | **Content** | **SEPA+PrEP** **Activities** |
| --- | --- | --- | --- |
| 1. | HIV in the Hispanic Community | - Basic facts about HIV among Hispanic women - Soap opera: “Sin Verguenza/ Without Shame” - What are HIV and AIDS? - How is HIV transmitted? - Introduction to Hispanic cultural values, beliefs, and traditions - Introduction to HIV prevention with an emphasis on PrEP | 1. Introduction and group commitments^a^ 2. Soap opera discussion^a^ 3. Facilitated small group discussions^a,b,c^ 4. Interactive activities^a,b,c^ 5. Home-based activities^a,b,c^ 6. Session evaluation^a,b,c^ 7. Skill-building exercises to promote condom use^b^ 8. Skill-building activities to communicate more effectively with partners and family^c^ 9. Role-playing^c^ 10. Intervention evaluation^c^ 11. Reflection and commitments^c^ 12. Certificate in recognition of SEPA+PrEP completion^c^ |
| 2. | HIV Prevention | - Rumors, myths, and beliefs surrounding HIV/AIDS - STIs and their role in HIV - HIV prevention   - PrEP  - HIV testing  - Condom use   - How Hispanic cultural values and traditions are related to HIV prevention |  |
| 3. | Partner Communication  & Negotiation/  Intimate Partner Violence Prevention in the Context of HIV | - Partner communication and negotiation strategies to prevent HIV - Self-esteem in the context of partner communication and negotiation - Intimate partner violence prevention in the context of HIV - HIV prevention discussion with an emphasis on review of PrEP content |  |

*Note.* AIDS = acquired immunodeficiency syndrome; HIV = human immunodeficiency virus;

STI = sexually transmitted infections; PrEP = Pre-Exposure Prophylaxis

^a^ Activity conducted in Session 1

^b^ Activity conducted in Session 2

^c^ Activity conducted in Session 3
