# Supplementary material for: Feasibility and acceptability of SEPA+PrEP: An HIV prevention intervention to increase PrEP knowledge, initiation, and persistence among cisgender heterosexual Hispanic women
Source: PLoS One. 2024 Jan 2;19(1):e0296080. doi: 10.1371/journal.pone.0296080 (PMC10760780; doi:10.1371/journal.pone.0296080)
Supplement: S2 Table — (DOCX) [file pone.0296080.s003.docx]

**Table 2. SEPA+PrEP Participants’ Socio-Demographic Characteristics (N=44**)*.*

| Variables | *n* (%) or *M* (*SD*; range) |
| --- | --- |
| Age (in years) | 37 (8.5; 18-49) |
| Relationship status |  |
| Married | 19 (43.2) |
| Living with a male partner (not married) | 16 (36.4) |
| Single | 7 (15.9) |
| Divorced | 2 (4.5) |
| Years of education | 9.3 (4.0; 0-16) |
| Employment status |  |
| Employed | 29 (65.9) |
| Not employed | 18 (38.3) |
| Main occupation |  |
| Nurseries (plant care) | 9 (31.0) |
| Housekeeping | 7 (9.1) |
| Homemaker | 5 (11.4) |
| Other (e.g., student, teacher, social worker) | 23 (52.3) |
| Per capita income | $1,989.3 ($934.1; $400-$4,000) |
| Preferred language |  |
| Spanish | 41 (93.2) |
| English | 3 (6.8) |
| Place of birth |  |
| Mexico | 23 (52.3) |
| Guatemala | 8 (18.2) |
| El Salvador | 5 (11.4) |
| United States | 4 (9.1) |
| Dominican Republic | 2 (4.5) |
| Colombia | 1 (2.3) |
| Honduras | 1 (2.3) |
| Years living in the U.S. | 16.1 (8.4; 0-31) |
| Has health insurance (no) | 28 (63.6) |

*Note.* *M* = mean; *n* = number; *SD* = standard deviation.
