# Supplementary material for: Feasibility and acceptability of SEPA+PrEP: An HIV prevention intervention to increase PrEP knowledge, initiation, and persistence among cisgender heterosexual Hispanic women
Source: PLoS One. 2024 Jan 2;19(1):e0296080. doi: 10.1371/journal.pone.0296080 (PMC10760780; doi:10.1371/journal.pone.0296080)
Supplement: S3 Table — (DOCX) [file pone.0296080.s004.docx]

**Table 3. SEPA+PrEP HIV Prevention Behaviors Prior to Participation.**

| **Questions** | ***n* (%) or *M* (*SD*; range)** |
| --- | --- |
| Number of times tested for HIV | 4.2 (5.4; 0-30) |
| Ever tested for HIV (yes) | 39 (88.6) |
| Reasons for testing |  |
| Tested during prenatal care | 23 (52.3) |
| Recommended by health care provider | 17 (38.6) |
| Ability to get results in same day | 7 (15.9) |
| Ability to maintain confidentiality of results | 6 (13.6) |
| Condom use with vaginal sex in previous 3 months |  |
| Never | 31 (70.5) |
| Less than half of the time | 3 (6.8) |
| Half of the time | 3 (6.8) |
| More than half of the time | 2 (4.5) |
| Always | 5 (11.4) |
| Concerned about becoming infected with HIV (no) | 25 (56.8) |

*Note.* *M* = mean; *n* = number; *SD* = standard deviation.
