# Supplementary material for: Feasibility and acceptability of SEPA+PrEP: An HIV prevention intervention to increase PrEP knowledge, initiation, and persistence among cisgender heterosexual Hispanic women
Source: PLoS One. 2024 Jan 2;19(1):e0296080. doi: 10.1371/journal.pone.0296080 (PMC10760780; doi:10.1371/journal.pone.0296080)
Supplement: S4 Table — (DOCX) [file pone.0296080.s005.docx]

**Table 4. Acceptability of the SEPA+PrEP Intervention.**

| **Questions** | ***n* (%) or *M* (*SD*; range)** |
| --- | --- |
| Understanding of the information deliveredª | 4.3 (1.0; 1-5) |
| High/Very high | 39 (88.6) |
| Neutral | 3 (6.8) |
| Low/Very low | 2 (2.5) |
| Satisfaction with the interventionª | 4.9 (0.3; 4-5) |
| Satisfied/Very satisfied | 44 (100.0) |
| Neutral | 0 (0) |
| Dissatisfied/Very dissatisfied | 0 (0) |
| Degree of comfort with the methodologies usedª | 4.4 (1.0; 1-5) |
| High/Very high | 39 (88.6) |
| Neutral | 2 (4.5) |
| Low/Very low | 3 (6.8) |
| Satisfaction with the facilitatorª* | 4.9 (0.4; 3-5) |
| High/Very high | 42 (97.7) |
| Neutral | 1 (2.3) |
| Low/Very low | 0 (0.0) |
| Satisfaction with the information providedª | 4.9 (0.3; 4-5) |
| Satisfied/Very satisfied | 44 (100.0) |
| Neutral | 0 (0) |
| Dissatisfied/Very dissatisfied | 0 (0) |
| Satisfaction with the intervention activitiesª | 4.8 (0.4; 4-5) |
| Satisfied/Very satisfied | 44 (100.0) |
| Neutral | 0 (0) |
| Dissatisfied/Very dissatisfied | 0 (0) |
| Inclusion of topics that are relevant for women like them^b**^ | 2.8 (0.5;1-3) |
| Yes | 33 (78.6) |
| Neutral | 8 (19.0) |
| No | 1 (2.4) |
| Fulfillment of initial expectations^b^ | 2.9 (0.4; 1-3) |
| Yes | 40 (90.9) |
| Partially | 3 (6.8) |
| No | 1 (2.3) |
| Likely to participate in similar programs^b^ | 3.0 (0.0; 3) |
| Yes | 44 (100) |
| Neutral | 0 (0) |
| No | 0 (0) |
| Likely to recommend the intervention to friends^b^ | 3.0 (0.0; 3) |
| Yes | 44 (100) |
| Neutral | 0 (0) |
| No | 0 (0) |
| Acceptability of an online version of the intervention^b^ | 1.8 (0.9; 1-3) |
| Yes | 22 (50.0) |
| Neutral | 9 (20.5) |
| No | 13 (29.5) |

*Note*. *One participant did not answer this question. ** Two participants did not answer this question. *M* = mean; *n* = number; *SD* = standard deviation.

^a^ Range of possible scores = 1-5.

^b^ Range of possible scores = 1-3.
