# Supplementary material for: Feasibility and acceptability of SEPA+PrEP: An HIV prevention intervention to increase PrEP knowledge, initiation, and persistence among cisgender heterosexual Hispanic women
Source: PLoS One. 2024 Jan 2;19(1):e0296080. doi: 10.1371/journal.pone.0296080 (PMC10760780; doi:10.1371/journal.pone.0296080)
Supplement: S1 File — (PDF) [file pone.0296080.s006.pdf]

UNIVERSITY
OF MIAMI

University of Miami
Human Subject Research Office (M809)
1400 NW 10th Avenue, Suite 1200A
Miami, FL 33136

Ph.: 305-243-3195
Fax: 305-243-3328
www.hsro.med.miami.edu

INITIAL REVIEW APPROVAL

August 20, 2020

Rosina Cianelli
305-284-2147
rcianelli@miami.edu

On 8/19/2020, the IRB reviewed and approved the following submission:

Type of Review:	Initial Study
Title of Study:	SEPA-PrEP: A Promising HIV Prevention Strategy for Cisgender Hispanic Heterosexual Women (CHHW) to Access, Initiate and Sustain Use of PrEP”
Investigator:	Rosina Cianelli
IRB ID:	20200856
Funding:	National Institutes of Health (NIH);
Number of Subjects Approved	55
Consent Requirements	The IRB approved a Waiver of Documentation of Consent<br><br>Note to PI: "Written information describing the research is to be provided to the subject or the subject’s legally authorized representative." The IRB reviewer noted that the study’s participants are working women, employed in mainly rural work. Their educational levels are unlikely to support the assumption that they will recall, easily, what has been discussed. What is more, the ICF script contains contact information, including a phone number, if they wish to obtain additional information about the study.

Investigator Manual	You must follow the requirements listed in the Investigator Manual (HRP-103)
Documents Reviewed:	•ACESEnglish.doc.docx<br>•Complete CHHW grant.docx<br>•Eligibility-Screening form SEPA+PREP English-Spanish.docx<br>•FLYER English SEPA+PREP.docx<br>•HIV Risk Perception.docx

UNIVERSITY
OF MIAMI

University of Miami
Human Subject Research Office (M809)
1400 NW 10th Avenue, Suite 1200A
Miami, FL 33136

Ph.: 305-243-3195
Fax: 305-243-3328
www.hsro.med.miami.edu

	•IC Verbal Consent Baseline and Initial Focus Group SEPA+PREP.doc<br>•IC Verbal Consent Baseline, Discussion Group, and Final Focus Group SEPA+PREP.doc<br>•Machismo and Marianismo.docx<br>•Parner Table.docx<br>•Protocol SEPA+PREP (4).docx<br>•Risk reduction behavioral intention.docx<br>•Self Efficacy for Condom Use.docx<br>•Self-Esteem.docx<br>•Socio demographic.docx<br>•Verbal Consent Script for Pre-Screening Process SEPA+PREP.docx

Additional Conditions of Approval - Please Read

1. In conducting this study, you are required to follow the requirements listed in the Investigator Manual (HRP-103).

2. This approval is limited to the items in the submission referenced above.

3. Approval from the IRB is required before making any modifications to the research unless the modification is necessary to prevent a subject from experiencing imminent harm.

4. You must submit Reports of New Information as required in the UM Investigator Manual HRP- 103, Chapter 8.

5. You must obtain IRB approval of translated documents before using them.

6. Approval of this study does not expire. However, you must submit a Continuing Review Report when you are ready to close this study. As a reminder, you should close studies when:
   - You are no longer interacting or intervening with human subjects to collect data about them; and
   - You are no longer accessing private identifiable information.

Should you have any questions, please contact: Vivienne Carrasco, Manager, IRB, (phone: 305-243-6713; email: vcarrasco@med.miami.edu).

UNIVERSITY
OF MIAMI

University of Miami
Human Subject Research Office (M809)
1400 NW 10th Avenue, Suite 1200A
Miami, FL 33136

Ph.: 305-243-3195
Fax: 305-243-3328
www.hsro.med.miami.edu
